# Comparative Study of 0.5% Bupivacaine, 0.5% Ropivacaine, and 0.75% Ropivacaine With Fentanyl as a Continuous Intraoperative Epidural Infusion on Post-operative Analgesia

**DOI:** 10.7759/cureus.59477

**Published:** 2024-05-01

**Authors:** Shashaank Pandey, Sharmila Borkar, Jovita M Monteiro, Sherin Mathew, Divya Vernekar, Ombretta Barreto, Pillai Arun Gopinathan, Vivek G Pillai, A. Vijeth Kishan, Isaac Lalbiekthang Joute

**Affiliations:** 1 Department of Anesthesiology, KMC Digital Hospital, Maharajganj, IND; 2 Department of Anesthesiology, Goa Medical College, Bambolim, IND; 3 Department of ENT, Goa Medical College, Bambolim, IND; 4 Department of Maxillofacial Surgery and Diagnostic Sciences, College of Dentistry, King Saud Bin Abdulaziz University for Health Sciences, Riyadh, SAU; 5 Department of ENT, Sub District Hospital, Chicalim, IND

**Keywords:** sensory blockade, ropivacaine, fentanyl, epidural, bupivacaine, analgesia

## Abstract

Introduction

Persistent postoperative pain leads to impaired patient recovery and delays in discharge of patients. The aim was to compare the efficacy of 0.5% bupivacaine to two varying concentrations of ropivacaine, specifically 0.5% and 0.75%, along with fentanyl as a continuous epidural infusion in providing adequate pain relief for patients subjected to infraumbilical surgeries.

Materials and methods

A prospective randomized comparative study was carried out on 150 patients and was divided into three groups, namely group B, group R, and group RP. Group B indicates (0.5% bupivacaine), group R means (0.5% ropivacaine), and finally, group RP means (0.75% ropivacaine); the three groups had 50 patients each. Group B was administered an epidural infusion of bupivacaine at a concentration of 0.5%, group R was given 0.5% ropivacaine, and group RP was treated with 0.75% ropivacaine; all three groups included 40 mcg fentanyl. The duration of the motor and sensory blockade and the time needed for the first rescue analgesia after the stoppage of epidural infusion were assessed in all three groups. The data were statistically analyzed using the ANOVA, “post hoc Tukey,” and chi-square tests.

Results

Comparison of the duration of motor and sensory blockade among all three groups showed that group RP (0.75% ropivacaine with 2 mcg/cc fentanyl) had the longest duration of 328.8 and 406 minutes, and the difference was statistically significant (p < 0.001). Comparison of the time of stoppage of epidural infusion to the requirement of first rescue analgesia showed that the group that received 0.75% ropivacaine with 40 mcg fentanyl had the highest value of 258.6 minutes and was statistically significant (p < 0.001).

Conclusion

Epidural intraoperative infusion of 0.75% ropivacaine with fentanyl offers better postoperative pain relief as compared to both 0.5% bupivacaine and 0.5% ropivacaine with fentanyl.

## Introduction

Pain can be described as “an unpleasant sensory or emotional experience together with actual or potential tissue damage, or described in terms of such damage,” according to the International Association for the Study of Pain [1]. Postoperative pain is defined as a situation of tissue injury associated with muscle spasms after surgery [2]. Malek et al. reported that 18.5% of patients stated pain as the worst experience in the postoperative period, and 36% of patients reported pain as the most common complaint after surgery [3]. 

Efficient postoperative pain management is a critical component of the care of surgical patients [4]. Uncontrolled post-operative pain may further lead to the development of chronic pain with poor quality of life [5]. The benefits of effective postoperative pain control are better patient compliance, early mobilization, lower risk of deep vein thrombosis, and faster recovery with fewer chances of neuropathic pain development. Appropriate pain management can further reduce the duration of hospital stays, thus lowering the financial burden and increasing patient satisfaction [6-8].

For postoperative pain treatment, the most potent method among the various pain relief strategies is afferent neural blocking with local anesthetics. Other alternatives encompass the use of high-dose opioids, opioids delivered via the epidural route, patient-controlled opioid therapy, and NSAIDs [6].

Epidural anesthesia has the potential to reduce postoperative morbidity and mortality. It has rapid action, recovery, hemodynamic stability, and effective postoperative analgesia [9,10]. Patient-controlled epidural analgesia has more benefits than intravenous (IV) controlled analgesia, which includes superior analgesia and suppression of stress response [11-13]. Prolonged postoperative pain causes a neuroendocrine stress response of protein catabolism, hyperglycemia, and increased oxygen demand [14].

The addition of opioids to local anesthetics has numerous advantages, including enhanced dynamic pain relief, limited regression of sensory blockade, and reduced doses of local anesthetic [15,16]. The rapid onset of action and clearance of fentanyl help prevent delayed respiratory depression [17]. Ropivacaine has several actions, like poor solubility in lipids, easy reversibility after IV injection, reductions in CNS complications, lesser motor block, and greater differentiation of sensory and motor block [10]. Compared to bupivacaine, ropivacaine exhibits a comparatively delayed initiation, diminished strength, reduced motor block duration, and a lesser likelihood of inducing complications in the cardiac and central nervous systems [18].

Although there are several studies conducted to compare bupivacaine and ropivacaine as a continuous epidural infusion, there are limited studies conducted to compare 0.5% bupivacaine, 0.5% ropivacaine, and 0.75% ropivacaine with 2 mcg/cc fentanyl as a continuous epidural infusion on post-operative analgesia. Hence, the study aimed to (i) draw a comparison between 0.5% bupivacaine, 0.5% ropivacaine, and 0.75% ropivacaine with 2 mcg/cc fentanyl as a continuous epidural infusion on post-operative analgesia and (ii) evaluate the quality of sensory and motor blockade of epidural infusion along with variations in blood pressure & heart rate.

## Materials and methods

A protocol for a prospective randomized trial was presented and approved by the Institutional Ethical Committee of the Office of the Department of Pharmacology under the Goa Government Medical College, Bambolim, India. A sample size of 150 patients who underwent infraumbilical surgeries in a supine position was decided by the statistician based on previous studies [19,20]. A written informed consent from patients and their relatives was taken. The study was conducted from October 2018 to March 2020 at the Department of Anesthesia.

The inclusion criteria were physical status according to the American Society of Anesthesiologists (ASA 1 and ASA 2), age between 18 years and 60 years, infra-umbilical surgeries of duration more than two hours under combined epidural spinal anesthesia, and body weight with a normal range of BMI (18.5-24.9). The exclusion criteria were hypersensitivity to local anesthetics, pregnant/lactating females, coagulation abnormality, and local site infection. Proper preoperative evaluation and clinical examination, along with investigations, were done.

The study sample was divided into three groups, with 50 patients in each group (randomization technique was followed). The three groups were the following: (1) group B: 3.0 mL 0.5% (H) bupivacaine intrathecally followed by epidural infusion of 0.5% bupivacaine with fentanyl (2 mcg/cc), (2) group R: 3.0 mL 0.5% (H) bupivacaine intrathecally followed by epidural infusion of 0.5% ropivacaine with fentanyl (2 mcg/cc), and finally, (3) group RP: 3.0 mL 0.5% (H) bupivacaine intrathecally followed by epidural infusion of 0.75% ropivacaine with fentanyl (2 mcg/cc).

Anesthesia procedure

The operation theater was prepared for both general and regional anesthesia. IV access was achieved under strict asepsis. The conventional monitors, as per ASA guidelines, were attached, and baseline values were recorded. Combined spinal-epidural (CSE) was then performed in patients under strict aseptic precautions.

Combined spinal-epidural blockade

The patient was placed on the operating table in a sitting position. The patient’s back was cleaned with 10% povidone-iodine solution and spirit and later draped with a sterile towel. L2-L3 space was chosen to perform epidural catheterization, and L3-L4 space subarachnoid blockade was done. The epidural was first performed. The epidural catheter was threaded, and the tip was placed 5 cm in the cephalad direction. The catheter was later secured.

L3-L4 spaces were identified, and the spinal blockade was performed using a 25 G spinal needle. After the free flow of CSF, 3.0 cc of 0.5% heavy bupivacaine was administered at a rate of 0.2 mL/sec. Immediately, the patient was put in a supine position. After one hour, epidural infusion of 0.5% bupivacaine with fentanyl (2 mcg/cc) at 6 cc/h (group B), epidural infusion of 0.5% ropivacaine with fentanyl (2 mcg/cc) at 6 cc/h (group R), and epidural infusion of 0.75% ropivacaine with fentanyl (2 mcg/cc) at 6 cc/h (group RP) were started.

Sensory blockade was evaluated by a pinprick test, while motor blockade was analyzed by a modified Bromage scale. Throughout the intraoperative period, pulse rate, non-invasive blood pressure (systolic and diastolic blood pressure), pulse oximetry (SPO2), and continuous electrocardiogram were recorded. Side effects like hypotension, bradycardia, nausea, vomiting, shivering, pruritus, and urinary retention were listed. The heart rate and blood pressure changes were observed at various intervals of surgery. Duration of surgery and total duration of epidural infusion were noted at the end of surgery.

Post-operative monitoring

**Figure 1:**
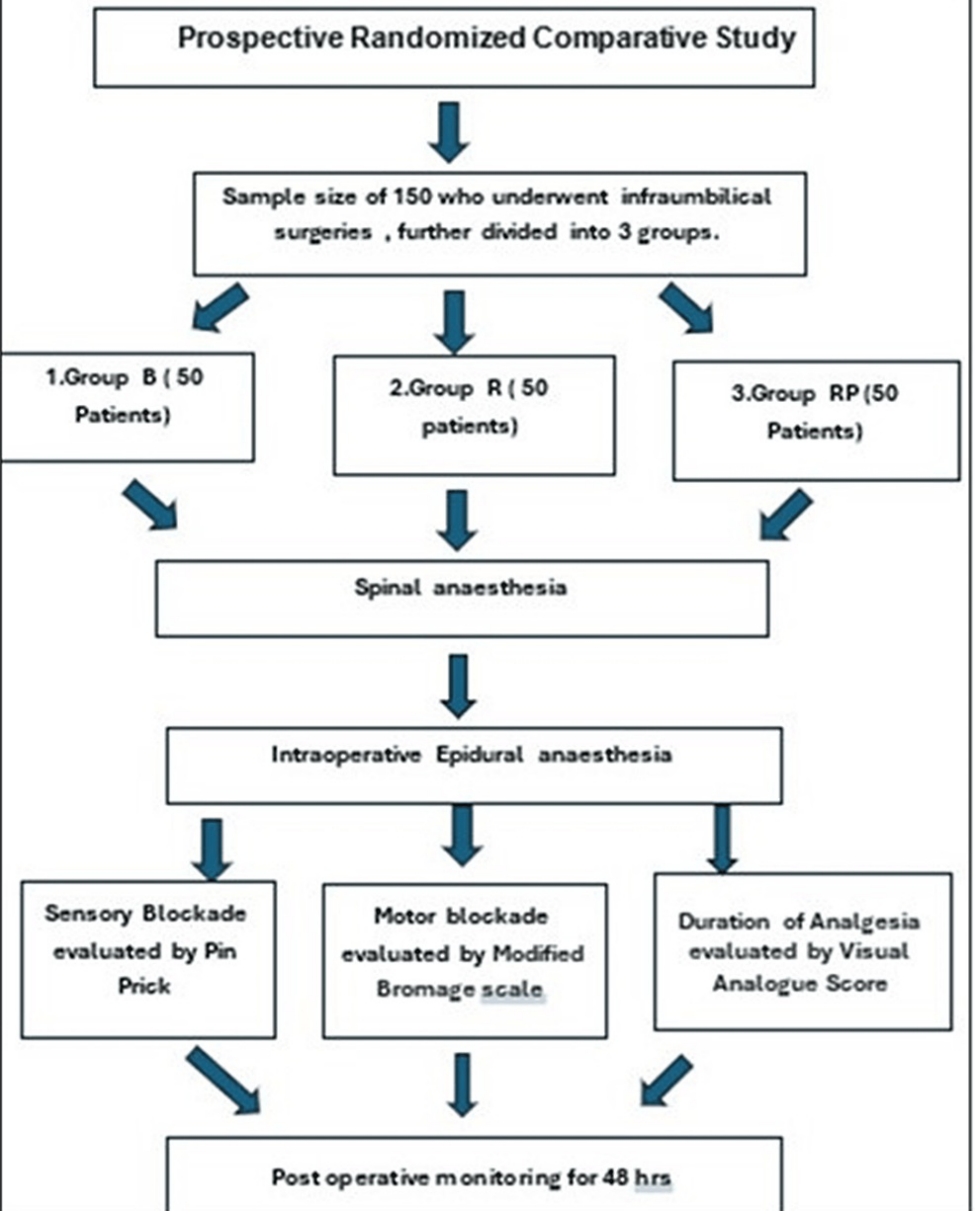
Consort chart showing the methodology

After surgery, patients were transferred to the post-anesthesia care unit. The duration of sensory and motor blockade, as well as the duration of analgesia, were monitored at regular intervals for 24 hours. The sensory block duration is defined as the period from when the subarachnoid drug is administered until the sensation in the heel and sole of the foot returns, indicating that the L5-S1 level has been reached. The motor block duration is defined as the period from when the drug is administered into the subarachnoid space until the patient reaches a score of 6 on the modified Bromage scale [21] (Table 1). The duration of analgesia was evaluated as the period from the moment of suspension of epidural infusion was stopped until the patient required the first postoperative analgesic or when the visual analog score [22] exceeded 5, or whichever came first. The study was concluded with respect to analgesia at that point (the summary of methodology is shown in Figure 1).

**Table 1:** Modified Bromage score (intensity of motor block) [21]

Score	Criteria
1	Complete block (unable to move feet or knees)
2	Almost complete block (able to move feet only)
3	Partial block (just able to move knees)
4	Detectable weakness of hip flexion (between scores 3 and 5)
5	No detectable weakness of hip flexion while supine (full flexion of knees)
6	Able to perform partial knee bend

Postoperatively, patients were observed for 48 hours, and later, the epidural catheter was removed. The data were submitted in Microsoft Excel and were analyzed with IBM SPSS Statistics, version 20.0 (IBM Corp., Armonk, NY, USA). The data were analyzed using the ANOVA, “post hoc Tukey,” and chi-square tests.

## Results

**Figure 2:**
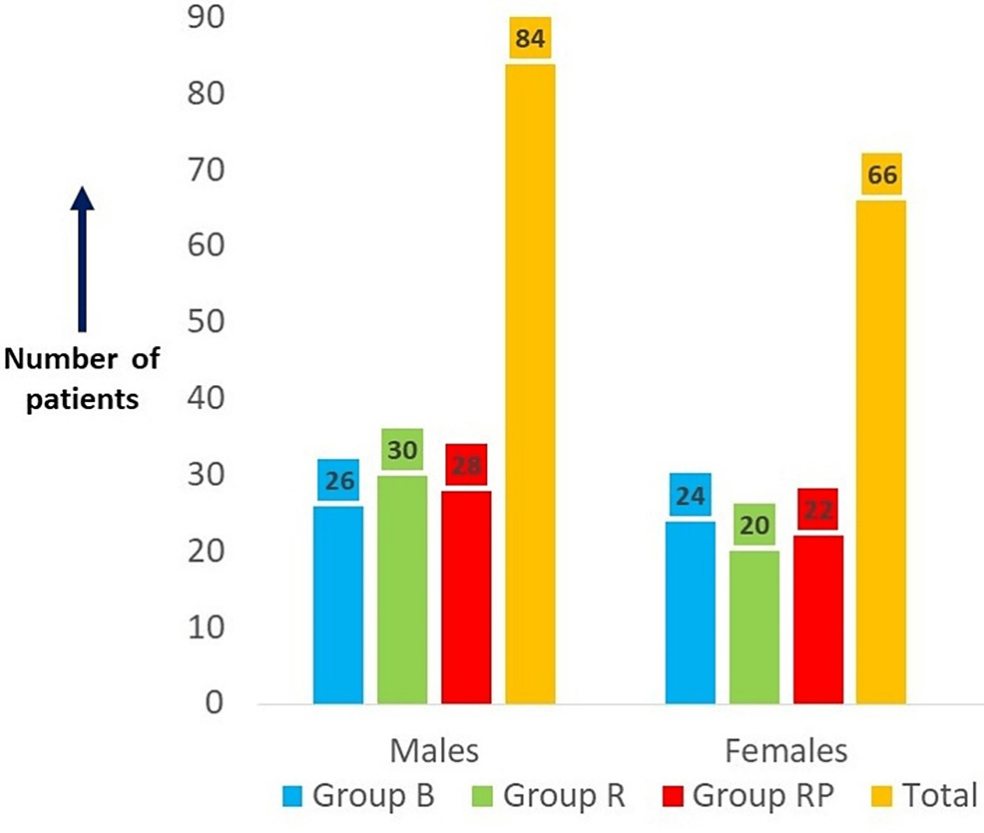
Gender distribution of the study participants across the three study groups Group B, 0.5% bupivacaine with 2 mcg/cc fentanyl; group R, 0.5% ropivacaine with 2 mcg/cc fentanyl; group RP, 0.75% ropivacaine with 2 mcg/cc fentanyl

A total of 150 patients divided into three groups (50 patients in each group) were considered. The three groups were group B (0.5% bupivacaine with 2 mcg/cc fentanyl), group R (0.5% ropivacaine with 2 mcg/cc fentanyl), and group RP (0.75% ropivacaine with 2 mcg/cc fentanyl). The comparison of age and gender between the three groups showed that the difference was not statistically significant (ANOVA = 0.794, p = 0.454; chi-square test = 0.649351, p = 0.723) (Table 2 and Figure 2). While the post hoc Tukey tests for age compared group B (0.5% bupivacaine with 2 mcg/cc fentanyl) and group R (0.5% ropivacaine with 2 mcg/cc fentanyl), group B (0.5% bupivacaine with 2 mcg/cc fentanyl) and group RP (0.75% ropivacaine with 2 mcg/cc fentanyl), and group R (0.5% ropivacaine with 2 mcg/cc fentanyl) and group RP (0.75% ropivacaine with 2 mcg/cc fentanyl), the differences were not statistically significant (p = 0.576, 0.478, and 0.986, respectively) (Table 2).

**Table 2:** Comparison of demographic data of age, height, and weight of patient characteristics Data = mean ± SD; S = significance; NS = not significant; SD = standard deviation Group B, 0.5% bupivacaine with 2 mcg/cc fentanyl; group R, 0.5% ropivacaine with 2 mcg/cc fentanyl; group RP, 0.75% ropivacaine with 2 mcg/cc fentanyl

Variables	Group B	Group R	Group RP	p-value	Comparison of group B & group R (post hoc Tukey test)	Comparison of group B & group RP (post hoc Tukey test)	Comparison of group R & group RP (post hoc Tukey test)
Age (mean) in years	41.14 ± 10.52	43.56 ± 12.35	43.94 ± 3.14	0.454 (NS)	-2.42 (NS)	-2.8 (NS)	-0.38 (NS)
Height (mean) in cm	160.24 ± 4.34	163.5 ± 4.9	160.32 ± 5.39	0.001 (S)	-3.26 (S)	-0.08 (NS)	3.18 (S)
Weight (mean) in kg	65.14 ± 4.34	64.48 ± 4.34	63.68 ± 4.34	0.265 (NS)	0.66 (NS)	1.46 (NS)	0.8 (NS)

When the comparison of height was done between the three groups, the difference was statistically significant (ANOVA = 7.219, p = 0.001) (Table 2). While post hoc Tukey tests for height compared group B (0.5% bupivacaine with 2 mcg/cc fentanyl) and group R (0.5% ropivacaine with 2 mcg/cc fentanyl) as well as group R (0.5% ropivacaine with 2 mcg/cc fentanyl) and group RP (0.75% ropivacaine with 2 mcg/cc fentanyl), the differences were statistically significant (p = 0.003 and 0.004, respectively). The comparison of group B (0.5% bupivacaine with 2 mcg/cc fentanyl) and group RP (0.75% ropivacaine with 2 mcg/cc fentanyl) was not statistically significant (p = 0.996) (Table 2).

Lastly, when weight was compared among demographic features between the three groups, the difference was not statistically significant (ANOVA = 1.339, p = 0.265) (Table 2). When the post hoc Tukey tests for weight were compared between group B (0.5% bupivacaine with 2 mcg/cc fentanyl) and group R (0.5% ropivacaine with 2 mcg/cc fentanyl), group B (0.5% bupivacaine with 2 mcg/cc fentanyl) and group RP (0.75% ropivacaine with 2 mcg/cc fentanyl) as well as group R (0.5% ropivacaine with 2 mcg/cc fentanyl) and group RP (0.75% ropivacaine with 2 mcg/cc fentanyl) were not statistically significant (p = 0.741, 0.235, and 0.644) (Table 2).

The comparison of time to reach the maximum sensory block (min) between the three groups shows that the difference was statistically significant (ANOVA = 13.982, p < 0.001). When the post hoc Tukey tests for maximum sensory block compared group B (0.5% bupivacaine with 2 mcg/cc fentanyl) and group R (0.5% ropivacaine with 2 mcg/cc fentanyl), the difference was not statistically significant (p = 1). Group B (0.5% bupivacaine with 2 mcg/cc fentanyl) and group RP (0.75% ropivacaine with 2 mcg/cc fentanyl) as well as group R (0.5% ropivacaine with 2 mcg/cc fentanyl) and group RP (0.75% ropivacaine with 2 mcg/cc fentanyl) groups when compared were statistically significant (p < 0.001) (Table 3).

**Table 3:** Summary of various parameters comparing the groups Data = mean ± SD; S = significance; NS = not significant; SD = standard deviation Group B, 0.5% bupivacaine with 2 mcg/cc fentanyl; group R, 0.5% ropivacaine with 2 mcg/cc fentanyl; group RP, 0.75% ropivacaine with 2 mcg/cc fentanyl

Parameters	Group B	Group R	Group RP	p-value	Group B vs group R (post hoc Tukey test)	Group B vs group RP (post hoc Tukey test)	Group R vs group RP (post hoc Tukey test)
Mean time required to reach maximum sensory block (min)	10.48 ± 0.58	10.48 ± 0.65	11.24 ± 0.92	<0.001 (S)	0 (NS)	-0.76 (S)	-0.76 (S)
Mean time required to reach maximum motor block (min)	5.48 ± 0.51	5.52 ± 0.58	5.64 ± 0.63	0.352 (NS)	-0.04 (NS)	-0.16 (NS)	-0.12 (NS)
Mean duration of surgery (min)	145.4 ± 16.81	148.2 ± 19.03	87.6 ± 18.36	0.718 (NS)	-2.8 (NS)	-2.2 (NS)	0.6 (NS)
Mean duration of epidural infusion (min)	85.4 ± 16.81	88.2 ± 19.03	87.6 ± 18.36	0.718 (NS)	-2.8 (NS)	-2.2 (NS)	0.6 (NS)
Mean duration of motor blockade (min)	283.2 ± 20.75	278.2 ± 23.53	328.8 ± 33.18	<0.001 (S)	5 (NS)	-45.6 (S)	-50.6 (S)
Mean duration of sensory blockade (min)	326.6 ± 24.71	341.2 ± 23.53	406 ± 32.33	<0.001 (S)	-14.6 (S)	-79.4 (S)	-64.8 (S)
Mean time of stoppage of infusion to requirement of first rescue analgesia	181.2 ± 13.8	193 ± 14.74	258.6 ± 17.5	<0.001 (S)	-11.8(S)	-77.4 (S)	-65.6 (S)

The comparison of the time to reach the max motor block between the three groups showed that the difference was not statistically significant (ANOVA = 1.051, p = 0.352). Post hoc Tukey tests compared group B (0.5% bupivacaine with 2 mcg/cc fentanyl) and group R (0.5% ropivacaine with 2 mcg/cc fentanyl), group B (0.5% bupivacaine with 2 mcg/cc fentanyl) and group RP (0.75% ropivacaine with 2 mcg/cc fentanyl), and group R (0.5% ropivacaine with 2 mcg/cc fentanyl) and group RP (0.75% ropivacaine with 2 mcg/cc fentanyl), the differences were not statistically significant (p = 0.935, 0.347, and 0.55, respectively) (Table 3).

A comparison of the duration of surgery between the three groups showed that the difference was not statistically significant (ANOVA = 0.332, p = 0.718). While post hoc Tukey tests for the duration of surgery compared group B (0.5% bupivacaine with 2 mcg/cc fentanyl) and group R (0.5% ropivacaine with 2 mcg/cc fentanyl), group B (0.5% bupivacaine with 2 mcg/cc fentanyl) and group RP (0.75% ropivacaine with 2 mcg/cc fentanyl), and group R (0.5% ropivacaine with 2 mcg/cc fentanyl) and group RP (0.75% ropivacaine with 2 mcg/cc fentanyl), the differences were not statistically significant (p = 0.72, 0.816, and 0.985, respectively) (Table 3).

The comparison of the duration of epidural infusion between the three groups showed that the difference was not statistically significant (ANOVA = 0.332, p = 0.718). While post hoc Tukey tests compared group B (0.5% bupivacaine with 2 mcg/cc fentanyl) and group R (0.5% ropivacaine with 2 mcg/cc fentanyl), group B (0.5% bupivacaine with 2 mcg/cc fentanyl) and group RP (0.5% ropivacaine with 2 mcg/cc fentanyl), and group R (0.5% ropivacaine with 2 mcg/cc fentanyl) and group RP (0.75% ropivacaine with 2 mcg/cc fentanyl), the differences were not statistically significant (p = 0.72, 0.816, and 0.985, respectively) (Table 3).

The comparison of the duration of motor blockade between the three groups showed that the difference was statistically significant (ANOVA = 42.851, p < 0.001). When post hoc Tukey tests compared group B (0.5% bupivacaine with 2 mcg/cc fentanyl) and group R (0.5% ropivacaine with 2 mcg/cc fentanyl), the difference was not statistically significant (p = 0.611). When group B (0.5% bupivacaine with 2 mcg/cc fentanyl) and group RP (0.75% ropivacaine with 2 mcg/cc fentanyl) as well as group R (0.5% ropivacaine with 2 mcg/cc fentanyl) and group RP (0.75% ropivacaine with 2 mcg/cc fentanyl) were compared, the difference showed statistical significance (p < 0.001) (Table 3).

The comparison of the duration of sensory blockade between the three groups shows that the difference was statistically significant (ANOVA = 121.273, p < 0.001). The post hoc Tukey tests compared all three groups, and the difference was statistically significant among all the groups with respect to the duration of sensory blockade (p = 0.222 and p < 0.001) (Table 3).

The comparison of the time of stoppage of infusion to the requirement of first rescue analgesia between all three groups showed that the difference was statistically significant (ANOVA = 365.376, p < 0.001). The post hoc Tukey tests compared all three groups with each other, and the difference was statistically significant among all the groups (p = 0.0001) with respect to the stoppage of infusion to the requirement of first rescue analgesia (Table 3).

**Figure 3:**
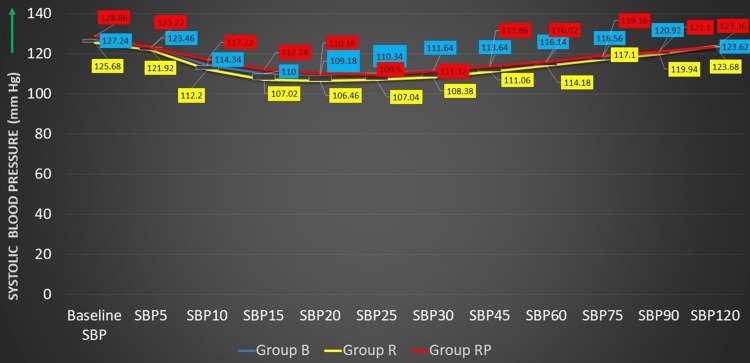
Distribution of study participants according to variations in SBP SBP, systolic blood pressure; group B, 0.5% bupivacaine with 2 mcg/cc fentanyl; group R, 0.5% ropivacaine with 2 mcg/cc fentanyl; group RP, 0.75% ropivacaine with 2 mcg/cc fentanyl

The comparison of baseline systolic blood pressure (SBP) between the three groups showed not much of a statistically significant difference in the variation of blood pressure; however, at 10 and 15 minutes, the variation in the three groups observed was found to be statistically significant (p < 0.05) (Figure 3 and Table 4).

**Table 4:** Distribution of study participants according to variations in SBP, DBP, and HR SBP, systolic blood pressure; DBP, diastolic blood pressure; HR, heart rate; group B, 0.5% bupivacaine with 2 mcg/cc fentanyl; group R, 0.5% ropivacaine with 2 mcg/cc fentanyl; group RP, 0.75% ropivacaine with 2 mcg/cc fentanyl

	Group B (n = 50)	Group R (n = 50)	Group RP (n = 50)	One-way ANOVA		Post hoc Tukey test		
				F-value (*=welch test)	p-value	Group B vs group R difference (p-value)	Group B vs group RP difference (p-value)	Group R vs group RP difference (p-value)
Baseline HR	84.54 ± 7.22	83.58 ± 6.91	82.48 ± 6.78	1.092	0.338	0.96 (0.771)	2.06 (0.305)	1.1 (0.711)
Baseline SBP	127.24 ± 8.37	125.68 ± 7.28	128.86 ± 8.3	1.976	0.142	1.56 (0.594)	-1.62 (0.57)	-3.18 (0.119)
Baseline DBP	77.9 ± 6.11	77.22 ± 6.09	76.92 ± 6.42	0.327	0.721	0.68 (0.848)	0.98 (0.71)	0.3 (0.968)
HR 5	78.78 ± 7.18	78.74 ± 6.93	80.16 ± 6.51	0.69	0.503	0.04 (1)	-1.38 (0.576)	-1.42 (0.558)
SBP 5	127.46 ± 8.27	121.92 ± 6.95	123.22 ± 8.95	0.523	0.594	1.54 (0.609)	0.24 (0.988)	1.3 (0.702)
DBP 5	74.94 ± 5.79	73.58 ± 5.44	71.92 ± 5.91	3.501	0.033	1.36 (0.461)	3.02 (0.025)	1.66 (0.317)
HR 10	70.66 ± 6.61	72.68 ± 6.63	77.98 ± 6.66	16.238	<0.001	-2.02 (0.283)	-7.32 (<0.001)	-5.3 (<0.001)
SBP 10	114.34 ± 8.05	112.2 ± 7.56	117.22 ± 11.15	3.865	0.023	2.14 (0.466)	-2.88 (0.253)	-5.02 (0.0017)
DBP 10	69.2 ± 4.95	67.36 ± 5.18	68.3 ± 7.31	1.212	0.3	1.84 (0.268)	0.9 (0.727)	-0.94 (0.706)
HR 15	69.18 ± 6.05	68.98 ± 6.73	76.08 ± 7.43	17.875	<0.001	0.2 (0.988)	-6.9 (<0.001)	-7.1 (<0.001)
SBP 15	110 ± 6.96	107.02 ± 7.33	112.24 ± 10.68	4.531*	0.013	2.98 (0.189)	-2.24 (0.387)	-5.22 (0.007)
DBP 15	66.54 ± 4.26	63.88 ± 4.87	65.62 ± 6.85	3.084	0.049	2.66 (0.041)	0.92 (0.675)	-1.74 (0.249)
HR 20	69 ± 6.49	67.78 ± 6.62	75.4 ± 6.88	18.854	<0.001	1.22 (0.632)	-6.4 (<0.001)	-7.62 (<0.001)
SBP 20	109.18 ± 6.96	106.46 ± 7.25	110.16 ± 9.03	3.034*	0.053	2.72(0.192)	-0.98 (0.805)	-3.7 (0.049)
DBP 20	66.02 ± 4.71	63.54 ± 5.39	64.68 ± 6.47	2.481	0.087	2.48 (0.07)	1.34 (0.454)	-1.14 (0.564)
HR 25	69.42 ± 5.74	67.86 ± 5.95	75.68 ± 6.27	23.866	<0.001	1.56 (0.396)	-6.26 (<0.001)	-7.82 (<0.001)
SBP 25	110.34 ± 6.93	107.04 ± 6.27	109.5 ± 8.13	2.876	0.06	3.3 (0.058)	0.84 (0.827)	-2.46 0.201
DBP 25	66.58 ± 4.13	64.36 ± 5.15	64.28 ± 6.27	3.085	0.049	2.22 (0.091)	2.3 (0.077)	0.08 (0.997)
HR 30	70.42 ± 5.96	68.32 ± 5.46	76.12 ± 6.03	24.031	<0.001	2.1 (0.172)	-5.7 (<0.001)	-7.8 (<0.001)
SBP 30	111.64 ± 6.97	108.38 ± 6.55	111.32 ± 8.38	2.995	0.053	3.26 (0.071)	0.32 (0.974)	-2.94 (0.115)
DBP 30	67.12 ± 4.36	64.8 ± 5.19	65.08 ± 5.59	3.114	0.047	2.32 (0.061)	2.04 (0.113)	-0.28 (0.959)
HR 45	71.24 ± 5.88	69.14 ± 5.06	77.2 ± 5.76	28.074	<0.001	2.1 (0.148)	-5.96 (<0.001)	-8.06 (<0.001)
SBP 45	113.64 ± 6.56	111.06 ± 5.98	113.86 ± 8.31	2.459	0.089	2.58 (0.161)	-0.22 (0.987)	-0.22 (0.987)
DBP 45	68.46 ± 4.51	66.32 ± 4.89	67.48 ± 6.58	1.967	0.144	2.14 (0.12)	0.98 (0.637)	-1.16 (0.532)
HR 60	72.26 ± 5.7	70.18 ± 4.92	78.08 ± 5.47	29.049	<0.001	2.08 (0.132)	-5.82 (<0.001)	-7.9 (<0.001)
SBP 60	116.14 ± 6.47	114.18 ± 5.8	116.02 ± 7.33	1.402	0.249	1.96(0.297)	0.12 (0.995)	-1.84 (0.342)
DBP 60	69.46 ± 3.81	68.14 ± 4.07	68.26 ± 6.16	1.157	0.317	1.32 (0.357)	1.2 (0.426)	-0.12 (0.991)
HR 75	73.22 ± 5.79	71.26 ± 5.02	78.8 ± 5.22	26.706	<0.001	1.96 (0.163)	-5.58 (<0.001)	-7.54 (<0.001)
SBP 75	118.56 ± 6.18	117.1 ± 5.35	119.16 ± 7.19	1.421	0.245	1.46 (0.478)	-0.6 (0.882)	-2.06 (0.233)
DBP 75	70.66 ± 3.17	69.66 ± 3.47	70 ± 5.71	0.71	0.493	(0.472)	0.66 (0.72)	-0.34 (0.916)
HR 90	73.78 ± 5.59	72.36 ± 5.29	79.44 ± 5.4	23.791	<0.001	1.42 (0.393)	-5.66 (<0.001)	-7.08 (<0.001)
SBP 90	120.92 ± 5.62	119.94 ± 4.48	121.1 ± 7	0.581	0.561	0.98 (0.674)	-0.18 (0.987)	-1.16 (0.578)
DBP 90	71.88 ± 3.53	71.52 ± 3.32	70.9 ± 4.93	0.768	0.466	0.36 (0.894)	0.98 (0.44)	0.62 (0.718)
HR 120	74.64 ± 5.4	73.3 ± 5	80.04 ± 5.05	23.968	<0.001	1.34 (0.397)	-5.4 (<0.001)	-6.74 (<0.001)
SBP 120	123.62 ± 5.65	123.68 ± 4.69	123.36 ± 6.64	0.044	0.957	-0.06 (0.998)	0.26 (0.972)	0.32 (0.958)
DBP 120	73.44 ± 3.77	73.16 ± 2.98	72.3 ± 4.31	1.271	0.284	0.28 (0.925)	1.14 (0.28)	0.86 (0.483)

**Figure 4:**
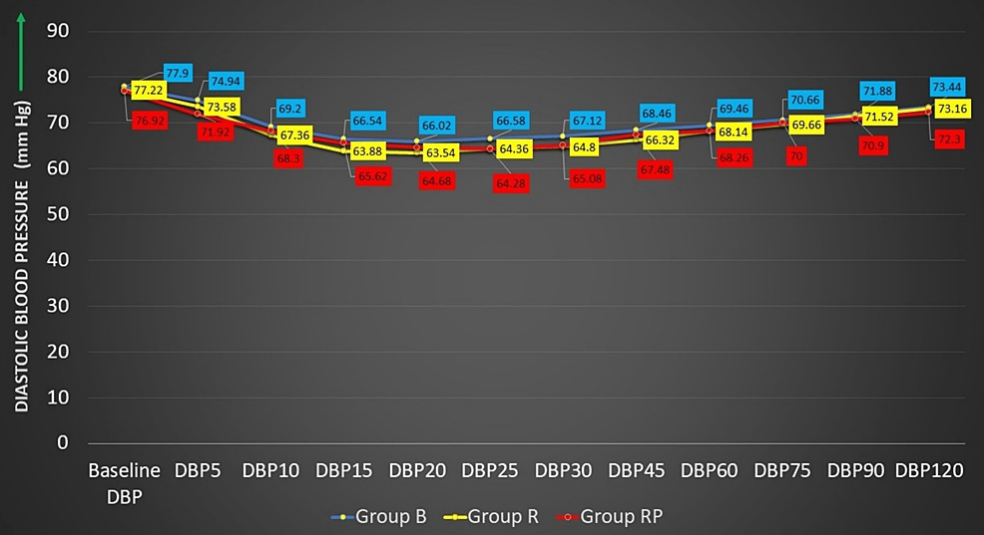
Distribution of study participants according to DBP DBP, diastolic blood pressure; group B, 0.5% bupivacaine with 2 mcg/cc fentanyl; group R, 0.5% ropivacaine with 2 mcg/cc fentanyl; group RP, 0.75% ropivacaine with 2 mcg/cc fentanyl

The comparison of baseline diastolic blood pressure (DBP) between the three groups showed that the difference was not statistically significant (test value = 0.327,p = 0.721). Statistically significant differences in the variation of DBP were observed at 5, 15, 25, and 30 minutes. The variation observed in the three groups was found to be statistically significant (p < 0.05) (Figure 4 and Table 4).

**Figure 5:**
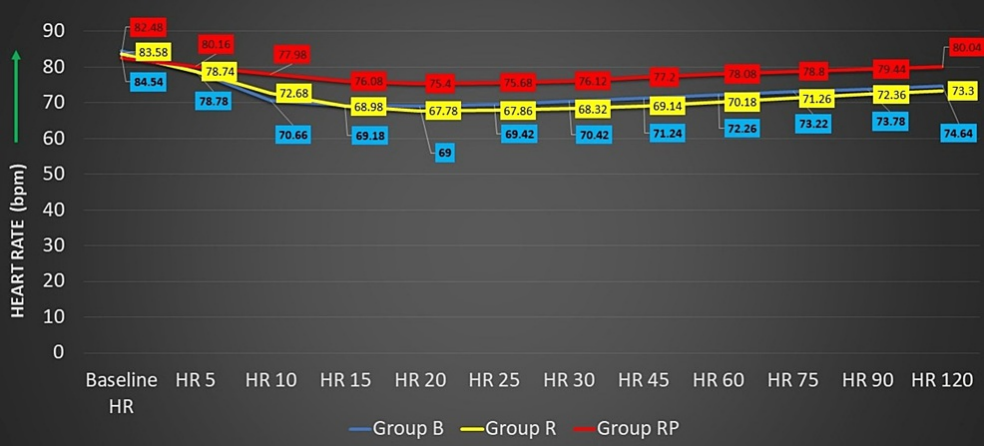
Distribution of study participants according to the variations in HR HR, heart rate; group B, 0.5% bupivacaine with 2 mcg/cc fentanyl; group R, 0.5% ropivacaine with 2 mcg/cc fentanyl; group RP, 0.75% ropivacaine with 2 mcg/cc fentanyl

The final comparison of baseline heart rate (HR) between the three groups showed that the difference was not statistically significant (test value = 1.092, p = 0.338). However, statistically significant variations (one-way ANOVA) in the HR were observed in comparison of three groups from 10 to 120 minutes (p < 0.05) (Figure 5 and Table 4).

## Discussion

Post-operative pain relief helps in the reduction of hospital stays, improves patient compliance, and helps in early mobilization post-surgery. The ideal pain management should have a long duration of action and fewer side effects [23]. Epidural analgesia is one of the most productive methods in post-operative pain management [24]. Studies have shown that epidural analgesia in combination with opioids is very effective [25]. Epidural analgesia has shown a reduction in complications, such as respiratory and cardiac morbidity, thromboembolic episodes, and gastrointestinal complications [25,26].

In the present study, a comparison of time to reach max sensory block (min) between the three groups shows that group RP (0.75% ropivacaine with 2 mcg/cc fentanyl) had the highest value of 11.24, group B (0.5% bupivacaine with 2 mcg/cc fentanyl) had the lowest value of 10.48, and the difference was statistically significant. A comparative study by Bindra et al. found that the onset of sensory block was 16.95, 19.70, and 22.35 minutes in groups 2, 3, and 1, respectively [27]. In a study similar to the one conducted by Finucane et al., the effects of three different doses of ropivacaine (0.5%, 0.75%, and 1%) were compared with 0.5% bupivacaine [19].

In this study, a comparison of time to reach max motor block (min) between the three groups shows that group RP (0.75% ropivacaine with 2 mcg/cc fentanyl) had the highest value of 5.64, group B (0.5% bupivacaine with 2 mcg/cc fentanyl) had the least value of 5.48, and the difference was statistically not significant. These findings were similar to a study done by Brockway et al., where the effects of different concentrations of ropivacaine (0.5%, 0.75%, and 1%) were compared with bupivacaine (0.5% and 0.75%) in terms of motor block onset [28]. In a study conducted by Wolf et al. and Brown et al., it was revealed that the clinical efficacy of ropivacaine and bupivacaine was compared postoperatively [29,30]. Interestingly, they found no statistically significant difference in the onset of sensory or motor block between these two anesthetics [29,30]. The present study also compared the duration of surgery (min) and the duration of epidural infusion (min) between the three groups, and the difference observed was not statistically significant. Similar findings were reported by Lakshmi et al. [20].

The duration of motor blockade (min) in the present study between the three groups showed that group B (0.5% bupivacaine with 2 mcg/cc fentanyl) and group R (0.5% ropivacaine with 2 mcg/cc fentanyl) were not statistically significant, while group B (0.5% bupivacaine with 2 mcg/cc fentanyl) and group RP (0.75% ropivacaine with 2 mcg/cc fentanyl) as well as group R (ropivacaine with 2 mcg/cc fentanyl) and group RP (0.75% ropivacaine with 2 mcg/cc fentanyl) were statistically significant. Research conducted by Korula et al. demonstrated that the outcome on motor block strength and duration was comparable between 0.75% ropivacaine and 0.5% bupivacaine [31]. Also, clinically, the quality of the two drugs was indistinguishable.

The comparison of the duration of sensory blockade (min) between the three groups showed that the difference was statistically significant. Thus stating that the duration of sensory blockade with 0.75% ropivacaine with 2 mcg/cc fentanyl was significantly higher when compared to other groups. Similar findings were reported by Bindra et al. [27], stating that the sensory blockade duration was significantly longer with 0.75% ropivacaine compared to 0.5% ropivacaine and 0.5% bupivacaine. These observations align with the results of a study done by Concepcion et al. [32].

However, other studies stated that ropivacaine had a lower duration and less potent motor block than bupivacaine [29,30]. Bindra et al. reported that the motor blockade was less in patients who were administered with 0.5% ropivacaine than those given with 0.5% bupivacaine and 0.75% ropivacaine [27]. When comparing ropivacaine with bupivacaine, the results of Brockway et al., Katz et al., Wolff et al., Finucane et al., and Brown et al. showed no significant difference in the duration of sensory analgesia [19,27-30,33].

The level of maximum sensory block of the study participants across the three study groups was T10 14 (9.3%), T6 64 (42.7%), and T8 72 (48%). However, this was observed to be not statistically significant among the three groups. A similar study conducted by Katz et al. reported that the maximum sensory block ranged from T2 to L1 [33]. The median sensory block height was T4 for the group receiving ropivacaine and T5 for the bupivacaine group. Another study conducted by Brown et al. reported that the maximum sensory block by 0.75% of ropivacaine was T4 [30]. These variations can be attributed to the different volumes of drugs used in their studies.

A comparison of the time of stoppage of infusion to the requirement of first rescue analgesia between the three groups showed that the difference was statistically significant. Various studies have reported that the need for rescue analgesia was more with the ropivacaine group, but it was observed to be less with an additional opioid [34,35]. However, another study reported that the need for rescue analgesia revealed no significant difference post-operatively between the study groups [20].

In this study, the comparison of baseline SBP and DBP between the three groups observed was found to be statistically significant. A similar finding was reported in a study conducted by Lakshmi et al. about variations in the mean arterial pressure in both the groups receiving ropivacaine and bupivacaine; both the groups were comparable with each other with statistically significant variations at 5 and 30 minutes [28]. Wolff et al. observed that the 0.75% and 1% ropivacaine groups had more frequent reductions in SBP and DBP than the 0.5% ropivacaine and bupivacaine groups, but this difference was not statistically significant [29].

Finally, baselined HR in the present study on a comparison of three groups showed statistically significant variations from 10 to 120 minutes; however, bradycardia was not reported among any group. Similar outcomes were obtained by Kampe et al. [36]. Finucane et al. and Brown et al. revealed that both bupivacaine and ropivacaine groups showed similar changes in heart rate and blood pressure with reference to cardiovascular changes [19,30].

Limitations

The present study was a single-based center study, limiting the geographical distribution of study participants and relatively smaller sample size to draw inferences that can be attributed to the general population. Also, no standardized epidural infusion in terms of timing and stopping was done.

## Conclusions

Bupivacaine 0.5% with 2 mcg/cc fentanyl, 0.5% ropivacaine with 2mcg/cc fentanyl, and 0.75% ropivacaine with 2 mcg/cc fentanyl as continuous epidural infusion in major Infraumbilical surgeries provide satisfactory postoperative analgesia. An epidural infusion of 0.75% ropivacaine with fentanyl provides better postoperative pain relief as compared to 0.5% bupivacaine with fentanyl and 0.5% ropivacaine with fentanyl.

## References

[1] Kumar KH, Elavarasi P (2016). Definition of pain and classification of pain disorders. J Adv Clin Res Insights.

[2] Ceyhan D, Güleç MS (2010). Is postoperative pain only a nociceptive pain?. Agri.

[3] Málek J, Ševčík P, Bejšovec D, Gabrhelík T, Hnilicova M, Křikava I, Mixa V Postoperative Pain Management. Prague, Czech Republic: Mladá fronta.Third edition.

[4] Sharrock NE, Cazan MG, Hargett MJ, Williams-Russo P, Wilson PD Jr (1995). Changes in mortality after total hip and knee arthroplasty over a ten-year period. Anesth Analg.

[5] Kehlet H, Holte K (2001). Effect of postoperative analgesia on surgical outcome. Br J Anaesth.

[6] Kehlet H, Jensen TS, Woolf CJ (2006). Persistent postsurgical pain: risk factors and prevention. Lancet.

[7] Ramsay MA (2000). Acute postoperative pain management. Proc (Bayl Univ Med Cent).

[8] Cousins MJ, Bridenbaugh PO, Carr DB, Horlocker TT (1998). Modification of responses to surgery by neural blockade. Cousins and Bridenbaugh's Neural Blockade in Clinical Anesthesia and Pain Medicine.

[9] Bauer M, George JE 3rd, Seif J, Farag E (2012). Recent advances in epidural analgesia. Anesthesiol Res Pract.

[10] Stienstra R (2003). The place of ropivacaine in anesthesia. Acta Anaesthesiol Belg.

[11] Gambling DR, McMorland GH, Yu P, Laszlo C (1990). Comparison of patient-controlled epidural analgesia and conventional intermittent "top-up" injections during labor. Anesth Analg.

[12] Owen H, Kluger MT, Ilsley AH, Baldwin AM, Fronsko RR, Plummer JL (1993). The effect of fentanyl administered epidurally by patient-controlled analgesia, continuous infusion, or a combined technique of oxyhaemoglobin saturation after abdominal surgery. Anaesthesia.

[13] Liu SS, Allen HW, Olsson GL (1998). Patient-controlled epidural analgesia with bupivacaine and fentanyl on hospital wards: prospective experience with 1,030 surgical patients. Anesthesiology.

[14] Hurley R, Wu C (2010). Acute postoperative pain. Miller’s Anesthesia.

[15] Srivastava U, Rana SP, Kumar A (2008). Role of epidural anaesthesia and analgesia in reducing postoperative morbidity and mortality during major abdominal surgery. Indian J Anaesth.

[16] Cooper DW, Turner G (1993). Patient-controlled extradural analgesia to compare bupivacaine, fentanyl and bupivacaine with fentanyl in the treatment of postoperative pain. Br J Anaesth.

[17] Fischer RL, Lubenow TR, Liceaga A, McCarthy RJ, Ivankovich AD (1988). Comparison of continuous epidural infusion of fentanyl-bupivacaine and morphine-bupivacaine in management of postoperative pain. Anesth Analg.

[18] Kuthiala G, Chaudhary G (2011). Ropivacaine: a review of its pharmacology and clinical use. Indian J Anaesth.

[19] Finucane BT, Sandler AN, McKenna J (1996). A double-blind comparison of ropivacaine 0.5%, 0.75%, 1.0% and bupivacaine 0.5%, injected epidurally, in patients undergoing abdominal hysterectomy. Can J Anaesth.

[20] Lakshmi K, Kumari MP, Sunil R (2015). A comparison of the analgesic efficacy and safety of epidural bupivacaine with fentanyl and ropivacaine with fentanyl in abdominal surgery. Ain-Shams J Anaesth.

[21] Breen TW, Shapiro T, Glass B, Foster-Payne D, Oriol NE (1993). Epidural anesthesia for labor in an ambulatory patient. Anesth Analg.

[22] AlHareky M, AlHumaid J, Bedi S, El Tantawi M, AlGahtani M, AlYousef Y (2021). Effect of a vibration system on pain reduction during injection of dental anesthesia in children: a randomized clinical trial. Int J Dent.

[23] Ng HP, Nordström U, Axelsson K, Perniola AD, Gustav E, Ryttberg L, Gupta A (2006). Efficacy of intra-articular bupivacaine, ropivacaine, or a combination of ropivacaine, morphine, and ketorolac on postoperative pain relief after ambulatory arthroscopic knee surgery: a randomized double-blind study. Reg Anesth Pain Med.

[24] Brodner G, Mertes N, Van Aken H, Pogatzki E, Buerkle H, Marcus MA, Mollhoff T (1999). Epidural analgesia with local anesthetics after abdominal surgery: earlier motor recovery with 0.2% ropivacaine than 0.175% bupivacaine. Anesth Analg.

[25] Scott DA, Beilby DS, McClymont C (1995). Postoperative analgesia using epidural infusions of fentanyl with bupivacaine. A prospective analysis of 1,014 patients. Anesthesiology.

[26] Jayr C, Beaussier M, Gustafsson U (1998). Continuous epidural infusion of ropivacaine for postoperative analgesia after major abdominal surgery: comparative study with i.v. PCA morphine. Br J Anaesth.

[27] Bindra TK, Singh R, Gupta R (2017). Comparison of postoperative pain after epidural anesthesia using 0.5%, 0.75% ropivacaine and 0.5% bupivacaine in patients undergoing lower limb surgery: a double blind study. Anesth Essays Res.

[28] Brockway MS, Bannister J, McClure JH, McKeown D, Wildsmith JA (1991). Comparison of extradural ropivacaine and bupivacaine. Br J Anaesth.

[29] Wolff AP, Hasselström L, Kerkkamp HE, Gielen MJ (1995). Extradural ropivacaine and bupivacaine in hip surgery. Br J Anaesth.

[30] Brown DL, Carpenter RL, Thompson GE (1990). Comparison of 0.5% ropivacaine and 0.5% bupivacaine for epidural anesthesia in patients undergoing lower-extremity surgery. Anesthesiology.

[31] Korula S, George GM, Ipe S, Abraham SP (2011). Epidural anesthesia and post-operative analgesia for bilateral inguinal mesh hernioplasty: comparison of equipotent doses of ropivacaine and bupivacaine. Saudi J Anaesth.

[32] Concepcion M, Arthur GR, Steele SM, Bader AM, Covino BG (1990). A new local anesthetic, ropivacaine. Its epidural effects in humans. Anesth Analg.

[33] Katz JA, Knarr D, Bridenbaugh PO (1990). A double-blind comparison of 0.5% bupivacaine and 0.75% ropivacaine administered epidurally in humans. Reg Anesth.

[34] Casati A, Santorsola R, Aldegheri G (2003). Intraoperative epidural anesthesia and postoperative analgesia with levobupivacaine for major orthopedic surgery: a double-blind, randomized comparison of racemic bupivacaine and ropivacaine. J Clin Anesth.

[35] Patil SS, Kudalkar AG, Tendolkar BA (2018). Comparison of continuous epidural infusion of 0.125% ropivacaine with 1 μg/ml fentanyl versus 0.125% bupivacaine with 1 μg/ml fentanyl for postoperative analgesia in major abdominal surgery. J Anaesthesiol Clin Pharmacol.

[36] Kampe S, Diefenbach C, Kanis B, Auweiler M, Kiencke P, Cranfield K (2002). Epidural combination of ropivacaine with sufentanil for postoperative analgesia after total knee replacement: a pilot study. Eur J Anaesthesiol.

